# Electrochemical Synthesis of Polyaniline and Sheet-like Structure of Molybdenum Selenide (PANI@2D-MoSe_2_) Binary Composite for Solar Cell Applications

**DOI:** 10.3390/nano15050384

**Published:** 2025-03-01

**Authors:** Alagumalai Manimekalai, Vediyappan Thirumal, Jinho Kim, Bathula Babu, Kuppu Sakthi Velu

**Affiliations:** 1 Department of Physics, Mother Teresa Women’s University, Kodaikanal 624101, Tamilnadu, India; manimekalaphysics@gmail.com; 2 Department of Mechanical Engineering, Yeungnam University, Gyeongsan-si 38541, Gyeongbuk-do, Republic of Korea; 3 Department of Physics, School of Sciences and Humanities, SR University, Warangal 506371, Telangana, India; 4 School of Chemical Engineering, Yeungnam University, Gyeongsan-si 38541, Gyeongbuk-do, Republic of Korea

**Keywords:** electropolymerization, polyaniline, molybdenum disulfide, PANI@2D-MoSe_2_, counter electrode, dye-sensitized solar cell, efficiency

## Abstract

In this work, a promising material of polyaniline (PANI) and two-dimensional molybdenum diselenides consisting of a PANI@2D-MoSe_2_ binary composite was prepared by an electrochemical polymerization ethod. The as-prepared PANI@2D-MoSe_2_, the polymer covered in the sheet-like structure of 2D-MoSe_2_ surface morphologies, was observed through FE-SEM and HR-TEM studies. The SAED pattern of PANI@2D-MoSe_2_ was observed to be in an octahedral phase. The octahedral crystalline phase was also confirmed based on the XRD pattern. In addition, EIS studies of the PANI@2D-MoSe_2_ binary composite counter electrode (CE) revealed the highest electrical conductivity of 3.47 × 10^−4^ S/cm at room temperature. The DSSCs assembled the PANI@2D-MoSe_2_ CE, which amounted to a 7.38% efficiency. Pristine PANI, 2D-MoSe_2_, and Pt CEs exhibited efficiencies of 5.07%, 5.82%, and 6.61%. The PANI integrated with 2D (MoSe) combines influences of conductivity and stability for future energy conversion technologies.

## 1. Introduction

Dye-sensitized solar cells (DSSCs) are low in cost, easy to fabricate, and have superior efficiency compared to other devices [1,2,3]. DSSCs consist of a dye-sensitized TiO_2_ as a photo-anode and a polymer-based I^−^/I^3−^ redox couple. In most high-efficiency solar cells, Pt is used as the counter electrode (CE) because of its higher conductivity, electrocatalytic activity, and better efficiency compared to other CE materials [4]. Hence, the Pt electrode is used in the fabrication of DSSCs; however, some demerits, such as the corrosion of the counter electrode and the instability of liquid electrolyte medium, make Pt-based CEs very difficult to use, with 40% of the cost alone being spent on CE preparation [5]. Several research reports have involved developments in the fields of lowering the cost and gaining higher efficiency in CEs, such as through the use of carbon-based materials [6], polymers [7], and polymer-based composites [8]. Several polymer-based composites have been used as CEs in DSSCs.

In recent years, the PANI-TMCs of PANI-WS_2_, PANI-MoS_2_, and PANI-WSe_2_ composites were used as CEs for DSSCs [9,10,11]. Among the composites, the PANI@2D MoSe2 composite has several merits as a CE. Due to the positively charged PANI attached to the negatively charged MoSe_2_, it provides a better active surface area, excellent electrical and electrocatalytic properties, and gives better stability and efficiency to the DSSC device. Additionally, various methods were used in the preparation of PANI@2D MoSe_2_ composite CEs for DSSCs like hydrothermal [12], solvothermal [13], chemical [14], and electrochemical polymerization methods [15]. Comparatively, among these methods, electrochemical polymerization techniques are one of the best choices for energy conversion technologies, due to the merits of their facile and controllable synthesis, their uniform deposition onto an FTO glass plate, and the fact that the obtained composite appears to be several micrometers to nanometers in thickness.

In this work, the electrochemically polymerized, highly conductive PANI polymerized on the sheet-like structure of MoSe_2_. The surface morphologies of the as-prepared composite materials were examined using FE-SEM and HR-TEM image analysis, in which they appear to have polymer-covered sheet-like structures. Furthermore, the XRD pattern of the electropolymerized PANI@2D MoSe_2_ exhibited an octahedral structure. The FT-IR spectral analysis of the as-prepared composite consists of cyano groups connected to molybdenum hydroxide in the regions of 1645 cm^−1^ and 3450 cm^−1^. Additionally, EIS studies found that a better electrical conductivity of 4.56 × 10^−3^ S/cm was achieved at room temperature. The DSSCs were assembled with pristine PANI, 2D MoSe_2_, PANI@2D MoSe_2_, and Pt counter electrodes. Finally, compared to high-performance CEs, the electropolymerized PANI@2D MoSe_2_ composite CE results in 8.13% efficiency.

## 2. Materials and Methods

### 2.1. Materials

Fluorine-doped tin oxide (FTO) coated on glass plates (TEC7) (specifications: dimension: 1″ × 1″ × 2.2 mm, nominal FTO film thickness: 250 nm), acetone, isopropanol, titanium dioxide, aniline monomer, hydrochloric acid (HCl), platinum paste, selenide powder, and ethanol were purchased from the Sigma-Aldrich chemical company, India Pvt. Ltd. (Safdarjung Enclave, New Delhi, India). PEO, acetonitrile, iodine, potassium iodide, and HMImI ionic liquid were obtained from Alfa Aesar. An N719 dye sensitizer was purchased from solaronix chemical company in Switzerland. All chemicals and materials utilized in the synthesis and characterization of the composites were of analytical grade and used without further purification.

### 2.2. Hydrothermal Synthesis of Flower-like Two-Dimensional Molybdenum Selenide (2D MoSe_2_)

**Figure 1:**
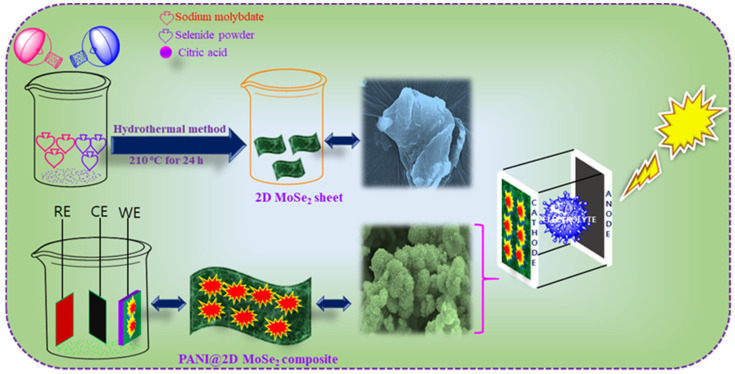
Scheme of 2D-MoSe_2_ sheet and PANI@2D-MoSe_2_ binary composite CE preparations for DSSCs.

Two-dimensional molybdenum selenide (2D-MoSe_2_) sheets were prepared using the hydrothermal method. First, 0.1 M of sodium molybdate, 0.1 M of selenide powder, and 0.1 M of citric acid were dispersed in 80 mL of deionized (DI) water for 30 min. The solutions were stirred for 4 h. Then, the above-mixed solutions were transferred into a 100 mL autoclave (Teflon-linked stainless steel). The reaction containing the Teflon-linked stainless steel was maintained at a temperature of 210 °C for 24 h. After 24 h, the reaction vessel cooled naturally. Then, the product solutions were washed with water and ethanol (centrifuged) several times. Finally, the precipitate was left to settle in the bottom of the centrifugation tube and then dried in a vacuum oven at 80 °C overnight. The dried, collected powder product was assessed and the two-dimensional sheet-like structure of 2D MoSe_2_ was obtained. The preparation and schematic diagram detailing the hydrothermal synthesis of the 2D MoSe_2_ counter electrode is presented in Figure 1.

### 2.3. Electrochemical Synthesis of Polyaniline and Two-Dimensional Molybdenum Selenide (PANI@2D MoSe_2_) Counter Electrode

The composite of PANI@2D MoSe_2_ was synthesized using the electrochemical polymerization method. Typically, 0.3 gm of MoSe_2_ is well dispersed in 50 mL of 01.M of HCl electrolyte solutions. The counter electrode (CE) is usually platinum (Pt), and the reference electrode (RE) is Hg/Hg_2_Cl_2_. Aniline monomer is dissolved in 0.1 molar of hydrochloric acidic electrolyte solution. The monomer concentration typically ranges from 0.1 M to ensure uniform polymerization. The scheme of the electrochemical synthesis of the PANI@2D MoSe_2_ composite counter electrode is shown in Figure 1.

### 2.4. Electropolymerization Parameters

The process was performed using cyclic voltammetry (CV), with the potential swept between −1.0 and 2.0 V at a scan rate of 50 mV/S for 10 cycles. A current density of 0.1–2.0 mA/cm^2^ was applied for controlled polymerization growth.

### 2.5. Device Fabrication

As-prepared PANI@2D MoSe_2_ was coated on an FTO plate using the doctor blade technique. Before the coating process, TiO_2_ NPs were soaked in 0.5 mM of ethanol containing N719 dye used as a photo-anode. In addition, 0.5 M of PEO and KI/I_2_ redox couples was used as the electrolyte medium. Then, 2D MoSe_2_, PANI, PANI@2D MoSe_2_, and Pt were coated on FTO plates and used as the CEs for the DSSC. The sandwich-type DSSC was assembled with N719 dye-sensitized TiO_2_ as the photo-anode and the PANI@2D MoSe_2_-coated FTO plate as the CE. The PEO containing KI/I_2_ redox couples was injected into the cell. Similar methods were used to fabricate DSSC devices with pristine 2D MoSe_2_, PANI, and Pt CEs.

### 2.6. Characterization

From FE-SEM and HR-TEM images, the surface morphology of 2D MoSe_2_, PANI, and PANI@2D MoSe_2_ was analyzed using an FEG Quanta 250 from the Czech Republic and an FEI Technai G2 F30 (FEI Company, Eindhoven, The Netherland) at magnifications ranging from 58× to 1× and an operating potential of 300 kV. The crystalline structure of PANI@2D MoSe_2_ was examined using an X-ray diffraction model ‘X’pert pro-PAN analytical diffractometer with Cu Kα radiation at 40 kV/30 mA. The functional groups of PANI@2D MoSe_2_ were identified via FT-IR spectral analysis, with spectra recorded using a Jasco 4600 (PS400, Jasco, Heckmondwike, UK) in the wavenumber range of 4000 cm^−1^ to 400 cm^−1^. The I–V performance of the dye-sensitized solar cell was measured using a solar simulator (Model 69907, Oriel, Newport, Irvine, CA, USA) and a Keithley source meter (2420) under AM1.5G illumination. A mask with a window of 0.15 cm^2^ was clipped onto the counter electrode side to define the active area of the cells.

## 3. Results and Discussion

### 3.1. Electrochemical Synthesis of PANI@2D-MoSe_2_ Composite

**Figure 2:**
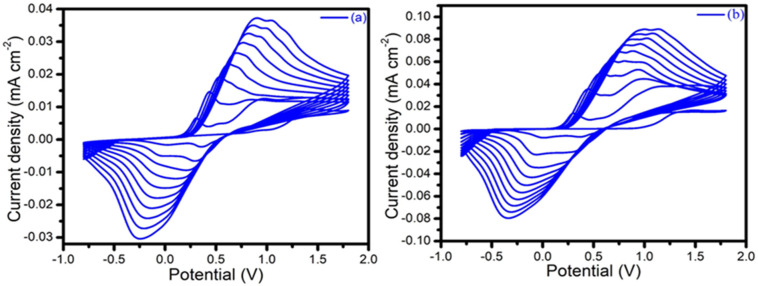
Electropolymerization curves of (**a**) neat PANI, (**b**) PANI@2D-MoSe_2_ composite.

During the electrochemical polymerization, characteristic peaks were observed in the voltammogram corresponding to the different redox states of the PANI@2D-MoSe_2_ composite. Figure 2a,b corresponded to CV curves of the PANI and PANI@2D MoSe_2_ composites. An anodic peak appeared at 0.3 V vs. Ag/AgCl, corresponding to the transition from the leucoemeraldine base (fully reduced form) to emeraldine salt (partially oxidized form). Another anodic peak was observed at 0.8 V vs. Ag/AgCl, indicating the transition from emeraldine salt to pernigraniline (fully oxidized form) [16].

The cathodic peaks in the range of −0.2 V to 0.6 V vs. Ag/AgCl were associated with the reduction of the emeraldine salt back to leucoemeraldine base and the reduction from pernigraniline to emeraldine salt.

**Electropolymerization steps:**

Oxidation of aniline monomers:

C_6_H_5_NH_2_ → C_6_H_5_NH_2_^+^ + e^−^

The aniline monomers are oxidized at the electrode surface, forming radical cations.

Dimerization of aniline radical cations:

2C_6_H_5_NH_2_^+^ → (C_6_H_4_NH_2_)_2_ + 2H^+^

The radical cations couple to form dimers, which further react to form long oligomers and, eventually, PANI chains.

Growth of PANI chains:

(C_6_H_4_NH_2_)_n_ + n e^−^ → (C_6_H_4_NH_2_)_n_ (PANI chains)

The PANI chains grow as the electropolymerization continues, forming a film on the MoSe_2_-coated FTO surface.

Electrochemical polymerization offers several advantages over solvothermal and chemical methods for synthesizing PANI@MoSe_2_. This technique enables the precise control of film thickness, morphology, and doping levels by adjusting the applied potential and deposition parameters. The approach encourages uniform polymer growth on MoSe_2_, which improves interfacial adhesion and facilitates charge transfer. Additionally, electrochemical polymerization occurs under mild conditions, eliminating the need for the harsh chemicals and high temperatures commonly required by other methods. This results in a highly conductive and defect-free polymer network, enhancing overall electrochemical performance. The method’s simplicity, reproducibility, and environmental friendliness make it an ideal choice for producing efficient PANI@MoSe_2_ composites for DSSCs.

### 3.2. FE-SEM Image Analysis

**Figure 3:**
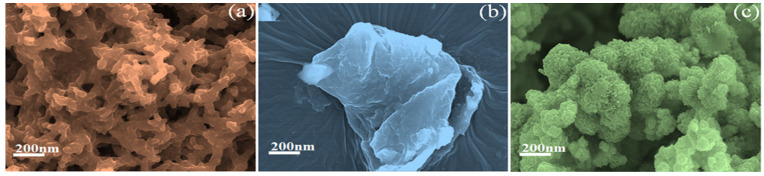
FE-SEM images of (**a**) PANI, (**b**) 2D-MoSe_2_, and (**c**) PANI@2D-MoSe_2_ composite.

Field-emission–scanning electron microscopy (FE-SEM) was used to analyze the surface morphologies of PANI, 2D-MoSe_2_, and PANI@2D-MoSe_2_. In the FE-SEM image analysis, the PANI surface morphology appeared in a nanofiber-like structure. The FE-SEM image of the PANI is depicted in Figure 3a. The 2D MoSe_2_ surface appears as a sheet-like structure in Figure 3b. An FE-SEM image of PANI@2D MoSe_2_ is shown in Figure 3c. The surface morphology of PANI@2D MoSe_2_ was observed in the PANI polymer covering a 2D MoSe_2_ sheet, and was also observed on uniform, smooth surfaces because the positive charge of the PANI polymer covalently connected to the negative charge of the molybdenum selenide [17]. The higher surface area of most of the surface edges of 2D MoSe_2_ resulted in the uniform deposition of PANI on the FTO plate. These combinations also led to better surface smoothness compared to PANI and 2D MoSe_2_. The FE-SEM imaging provided high-resolution morphological insights into the PANI@2D-MoSe_2_ composite, revealing its uniform distribution and nanoscale features. This helped in understanding the surface roughness, porosity, and interfacial interactions, which are crucial for enhancing charge transfer and catalytic activity in DSSCs.

### 3.3. HR-TEM Image Analysis

**Figure 4:**
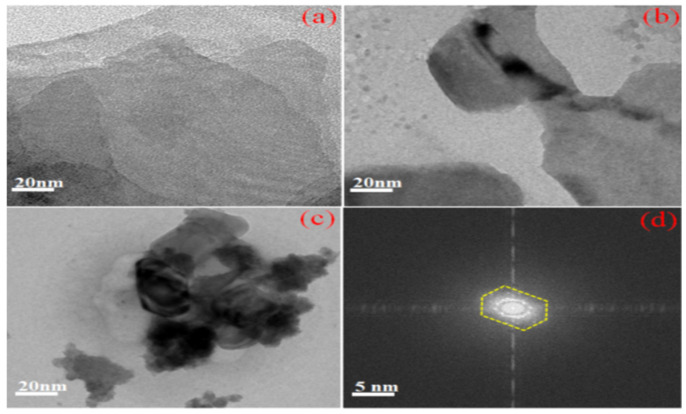
HR-TEM images of (**a**) PANI, (**b**) 2D MoSe_2_, (**c**) PANI@2D MoSe_2_, and (**d**) SAED pattern of PANI@2D-MoSe_2_ composite.

High-resolution transmission electron microscopy (HR-TEM) was used to explain the internal surface phenomenon of the PANI, 2D-MoSe_2_, and PANI@2D-MoSe_2_ composite counter electrodes. Figure 4a–c show the HR-TEM images of PANI, 2D MoSe_2_, and PANI@2D MoSe_2_. The surface morphology of PANI was found to have a nanofiber structure, as seen in Figure 4a. The 2D MoSe_2_ surface morphology appears as a sheet-like structure, as shown in Figure 4b. The electropolymerized PANI@2D MoSe_2_ surface morphologies were found to have a sheet-like structure. Here, the polymer was uniformly deposited on the 2D MoSe_2_. Due to polymers being deposited on the more active edges and the better surface active area of the metal selenides, these composites improve the crystalline growth and crystalline nature of the working electrode surface. An HR-TEM image of the electropolymerized PANI@2D MoSe_2_ is shown in Figure 4c [18]. The selected area electron diffraction (SAED) pattern of the as-prepared composite was observed in the hexagonal crystalline structure, as shown in Figure 4d. The octahedral crystalline structure of the PANI@2D-MoSe2 composite plays a crucial role in boosting its electrical conductivity, making it a strong candidate for use as a counter electrode in energy applications. The 2H-phase MoSe2, known for its octahedral coordination, exhibits metallic-like behavior due to the overlap of Mo-4d orbitals, which facilitates efficient charge transport. The addition of polyaniline (PANI) further enhances conductivity through π-π interactions and polaronic conduction, resulting in a synergistic effect between the two materials.

The composite’s layered design offers a significant surface area, increasing the number of active sites for charge transfer, which, in turn, lowers interfacial resistance and improves electron mobility. Additionally, the creation of a heterostructure enhances ion diffusion, making the composite particularly effective as a counter electrode in DSSCs. The combination of MoSe_2_ metallic conductivity and PANI’s redox-active properties results in exceptional electrocatalytic activity, reduced charge transfer resistance (Rct), and improved overall performance in energy storage and conversion systems. Finally, HR-TEM imaging offers atomic-scale resolution, enabling the visualization of the lattice fringes and crystallinity of the PANI@2D-MoSe_2_ composite. This helps confirm the intimate interaction between PANI and MoSe_2_, which is essential for efficient charge transfer and electrocatalytic performance in DSSCs.

### 3.4. XRD Pattern Analysis

**Figure 5:**
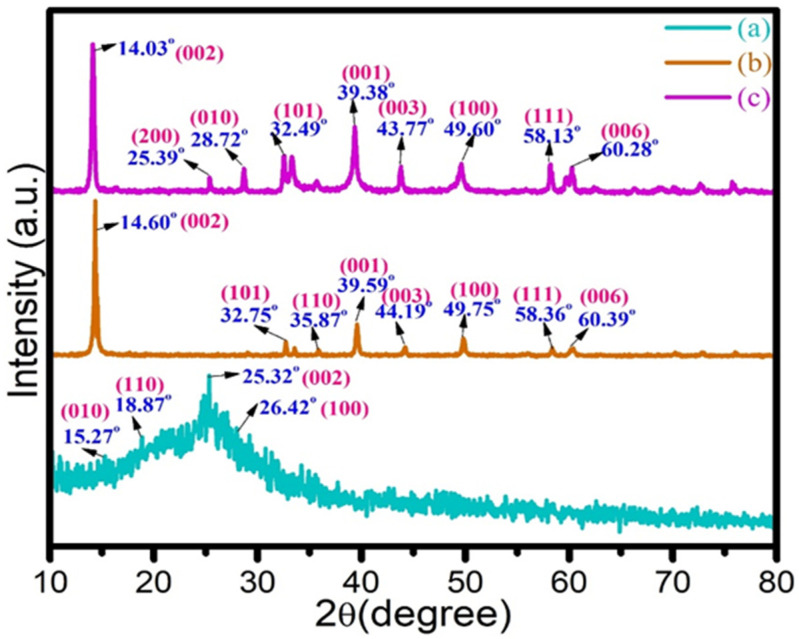
XRD patterns for (**a**) PANI, (**b**) 2D MoSe_2_, and (**c**) PANI@2D MoSe_2_ composite.

X-ray diffraction (XRD) pattern analysis was used to determine the crystalline nature of the PANI, 2D-MoSe_2_, and PANI@2D-MoSe_2_ composite. Figure 5a–c correspond to the XRD pattern of PANI, 2D MoSe_2_, and PANI@2D MoSe_2_. The XRD pattern of PANI (Figure 5b) appeared to be semi-crystalline in nature, with crystalline peaks observed at 2θ = 15.27°, 2θ = 18.87°, 2θ = 25.32°, and 2θ = 26.42°. These peaks also correlated to the crystalline planes of (010), (110), (002), and (100). The predominant peak at 2θ = 25.32° and the crystalline peak at (200) were associated with hydrogen bonds between polymer chains. The XRD pattern of 2D MoSe_2_ is shown in Figure 5b. Peaks appeared at 2θ = 14.60°, 32.75°, 35.87°, 39.59°, 44.14°, 44.75°, 49.75°, 58.36°, and 60.39°, corresponding to the 2D MoSe_2_, and crystalline planes were also observed at (002), (101), (110), (001), (003), (100), (111), and (006). The 2D MoSe_2_ XRD patterns exhibited octahedral structures. The peaks observed at 2θ = 14.03°, 25.39°, 28.72°, 32.49°, 39.38°, 43.77°, 49.60°, 58.13°, and 60.28° corresponded to the PANI@2D-MoSe_2_ composite, with crystalline planes also observed at (002), (200), (010), (101), (001), (003), (100), (111), and (006). Based on the XRD results, the polyaniline polymer was completely deposited on the MoSe_2_ sheets. The 2θ = 14.03°, 39.38°, and 60.28° peaks correspond to the 1-T phase of 2D MoSe_2_ in these XRD patterns, and the crystalline plane also appeared at (002), (101), (111), (103), (003), and (100). Figure 5c shows the XRD pattern for PANI@2D-MoSe_2_ [19,20]. During the electrochemical polymerization processes, the PANI polymer was completely inserted into the sheet-like structure of the two-dimensional MoSe_2_. After the insertion of polyaniline polymer into the molybdenum selenide, the semiconducting nature of the composites was reduced. The metallic nature of the octahedral phase was further increased and the crystalline nature of the as-prepared composites of PANI@2D-MoSe_2_ improved. The PANI and 2D MoSe_2_ were stacked with weak Van der Waals interactions between them.

The degree of crystallinity of PANI@2D-MoSe_2_ was calculated using Equation (1).

(1) $$Crystallinity=\frac{Area\;of\;the\;crystalline\;peaks}{Area\;of\;all\;peaks\;(Crystalline+Amorphous)}\times 100$$

The degrees of crystallinity of PANI, 2D-MoSe_2_, and PANI@2D-MoSe_2_ composite were 18.03%, 48.23%, and 62.04%.

We investigated whether PANI exists in a semi-crystalline state within the composite. The weak diffraction peaks in XRD typically indicate a more amorphous nature. The crystallinity affects charge transport: higher crystallinity can enhance carrier mobility, while amorphous regions may introduce charge-trapping sites. The XRD analysis identified the crystalline phases of the PANI@2D-MoSe_2_ composite, confirming structural integrity and phase purity. It also provided insights into interlayer spacing and crystallite size, which influence charge transport and catalytic activity in DSSCs.

### 3.5. FT-IR Spectral Analysis

**Figure 6:**
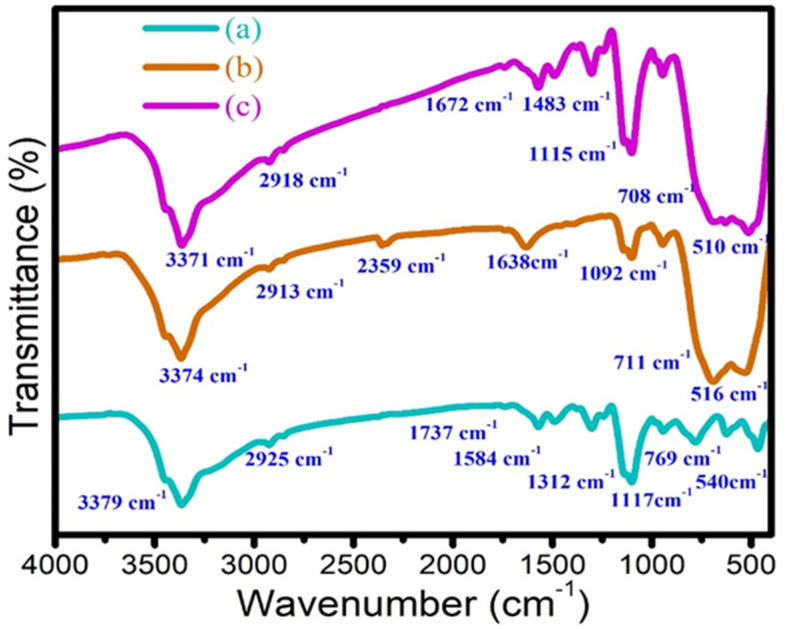
FT-IR spectra of (**a**) PANI, (**b**) 2D MoSe_2_, and (**c**) PANI@2D MoSe_2_ composite.

Fourier-transform infrared spectroscopy (FT-IR) is used to identify the presence of functional groups of the PANI, 2D-MoSe_2_, and PANI@2D-MoSe_2_ composite. The FT-IR spectra of the electropolymerized PANI are shown in Figure 6a. The peaks appeared at 3379, 2925, 1737, 1584, 1312, 1117, 769, and 540 cm^−1^, corresponding to the stretching vibrations of –NH_2_ and –CH_3_ groups, and the stretching vibrations of C=O, C-N, and -OH.

Figure 6b shows the FT-IR spectra of 2D MoSe_2_, with peaks at 3374, 2913, 2359, 1638, 1092, 711, and 516 cm^−1^, ascribed to the stretching vibrations of -OH, -CH_3_, C=O, C-O/OH groups, and metal selenide ions. The FT-IR spectra of PANI@2D MoSe_2_ are shown in Figure 6c. The peaks appeared at 510, 708, 1115, 1483, 1672, 2918, and 3371 cm^−1^, associated with the stretching vibrations of selenide ions, -NH_2_, C-N, -CH_3_, and –OH groups [21]. The peaks at 1672 cm^−1^ and 3371 cm^−1^ correspond to the stretching vibrations of cyano groups attached to molybdenum hydroxide. The peak at 3371 cm^−1^ in the FT-IR spectrum of the PANI@2D-MoSe_2_ composite, attributed to cyano groups attached to molybdenum hydroxide, can influence charge transfer in DSSCs. Cyano groups introduce localized electronic states that may enhance charge carrier mobility by facilitating electron delocalization and reducing recombination losses. Additionally, their interaction with molybdenum hydroxide can modulate the electronic structure of MoSe_2_, potentially improving its catalytic activity and interfacial charge transfer. This modification could contribute to the overall efficiency of the DSSC by optimizing electron transport dynamics. In addition, FT-IR spectroscopy confirms the functional groups in the PANI@2D-MoSe_2_ composite, ensuring successful polymerization and interaction between PANI and MoSe_2_. It also provides insights into chemical bonding, which influences charge transfer and electrochemical performance in DSSCs.

### 3.6. Electrochemical Impedance Spectroscopy (EIS) Studies

**Figure 7:**
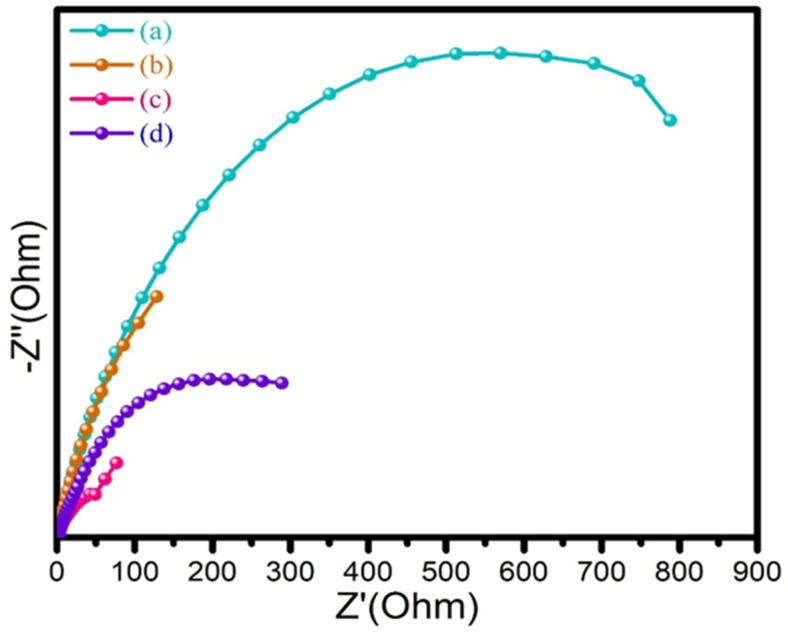
EIS curves of (**a**) PANI, (**b**) 2D MoSe_2_, (**c**) PANI@2D MoSe_2_, and (**d**) Pt CEs.

The electrical conductivity of the as-prepared PANI, 2D-MoSe_2_, and PANI@2D-MoSe_2_ composites were determined using electrochemical impedance spectroscopy (EIS) studies. EIS studies were used to determine the electrical conductivity of PANI, 2D MoSe_2_, and PANI@2D MoSe_2_ composite CEs. Figure 7a illustrates the EIS spectrum of electropolymerized PANI, showing the higher semicircle portion. This kind of semicircle portion indicates lower electrical conductivity. Figure 7b shows the EIS spectrum of 2D MoSe_2_, with Nyquist plots showing the higher semicircle portions. Figure 7c shows the EIS spectrum of the PANI@2D MoSe_2_ CE, which exhibits a lower semicircle portion and lower-frequency regions.

The lower semicircle portion and lower-frequency regions of the CE indicate lower charge transfer resistance, which results in higher electrical conductivity. Therefore, the as-prepared PANI@2D MoSe_2_ composite CE shows a lower semicircle portion, indicating lower charge transfer resistance and higher electrical conductivity compared to pristine 2D MoSe_2_, PANI, and Pt. This is due to the PANI NFs being covered on the 2D MoSe_2_ sheets. The Pt CE-based DSSCs and their EIS curves are shown in Figure 7d, also exhibiting a higher-frequency region, higher semicircle portion, and lower electrical conductivity compared to PANI@2D MoSe_2_. This composite has more surface active edges, uniform deposition of PANI on 2D MoSe_2_ sheets, and better adhesion between the CE and electrolyte interface. The PANI@2D MoSe_2_ composite counter electrode exhibited better electrical conductivity [12,22]. EIS studies revealed that the PANI@2D-MoSe_2_ composite exhibits superior electrical conductivity compared to PANI and 2D-MoSe_2_. In the Nyquist plot, the semicircle in the high-frequency region represents the charge transfer resistance (R_ct_) at the electrode/electrolyte interface. A smaller semicircle indicates lower Rct, signifying faster charge transfer and enhanced conductivity. The PANI@2D-MoSe_2_ composite shows the smallest semicircle, with an Rct value of 3.14 ohm cm^2^, confirming its excellent charge transport properties. This can be attributed to the uniform deposition of polyaniline on two-dimensional MoSe_2_ sheets, which enhances electron mobility, increases surface area, and improves electrocatalytic activity for efficient redox reactions. EIS analysis evaluates the charge transfer resistance and conductivity of the PANI@2D-MoSe_2_ composite, helping to understand its electrochemical performance. A lower charge transfer resistance indicates efficient electron transport, enhancing DSSC efficiency.

### 3.7. I–V Characterization

**Figure 8:**
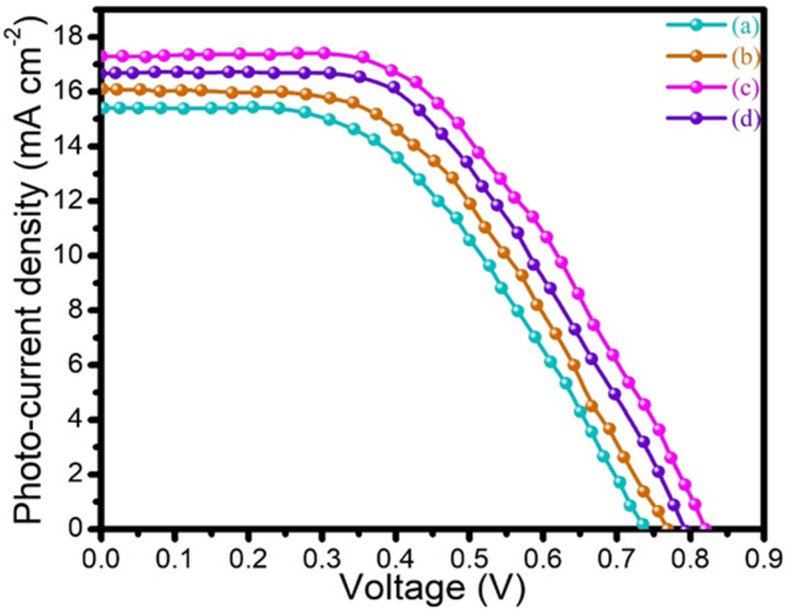
I–V curves of (**a**) PANI, (**b**) 2D MoSe_2_, (**c**) PANI@2D MoSe_2_, and (**d**) Pt CEs.

The I–V characterization curves of the PANI, 2D MoSe_2_, PANI@2D MoSe_2_, and Pt CEs are presented in Figure 8a–d and the measurement values are listed in Table 1. The efficiency of the solar cell devices was calculated using the following formulae:

FF = (J_max_ × V_max_)/(J_sc_ × V_oc_) (2)

ƞ (%) = (J_sc_ × V_oc_ × FF)/P_in_ × 100 (3)

Here, J_max_ is the maximum current output, V_max_ is the maximum voltage output, J_sc_ is the short-circuiting current density, V_oc_ is the open circuit voltage, FF is the fill factor, and η is the efficiency.

The I–V curves of the PANI NF and 2D MoSe_2_ sheet CEs are presented in Figure 8a,b. The DSSC device fabricated with the PANI NF CE achieved 5.07% efficiency, while the 2D MoSe_2_ sheet CE achieved an efficiency of 5.82%. The DSSC device using the Pt CE achieved an efficiency of 6.61%. The PANI@2D MoSe_2_ CE achieved a higher efficiency of 7.38% due to the higher electrical conductivity of the PANI covering the metallic nature of the octahedral phase of the 2D MoSe_2_. The carbon-based materials of the PANI combined TMCs, further improving the surface active area of CE and providing better interfacial contact between the CE and electrolyte interface, faster regeneration of iodide/tri-iodide redox couple, and reducing the recombination rates of the CE. The increasing current and potential of the device increased the fill factor of the device. Increasing the fill factor of the device always increases the efficiency of the device. The I–V curves of the PANI@2D MoSe_2_ and Pt CEs are presented in Figure 8c,d [23,24].

The WO_3_@PANI composite achieved an efficiency of 6.78% with a charge transfer resistance (R_ct_) of 10 ohm cm^2^ [19]. The poly(pyrrole) (PPy) polymer attached to the strontium titanate (PPy-SrTiO_3_) composite counter electrode exhibited a lower efficiency of 2.52% and a higher R_ct_ value of 46.4 ohm cm^2^ [25]. The nickel–polyaniline–graphene-based composite counter electrode demonstrated an efficiency of 5.80% with an R_ct_ of 10.78 ohm cm^2^ [26]. Similarly, the PANI-3% cobalt sulfide composite counter electrode achieved an efficiency of 6.14% with an R_ct_ value of 12.02 ohm cm^2^ [27]. Among these, the as-prepared PANI@2D-MoSe_2_ composite exhibited the highest efficiency of 7.38% and the lowest Rct value of 3.14 ohm cm^2^. The superior performance of the electropolymerized PANI@2D-MoSe_2_ composite can be attributed to the uniform deposition of polyaniline on the two-dimensional molybdenum selenide sheets. This sheet-like structure offers a higher surface area, improved electrical conductivity, and enhanced electrocatalytic activity due to the presence of selenide materials. Additionally, it facilitates the rapid regeneration of the iodide/tri-iodide redox couple, contributing to its enhanced efficiency.

Finally, the improved efficiency of 7.38% in DSSCs with the PANI@2D-MoSe_2_ composite can be attributed to several key factors. The strong interfacial interaction between PANI and MoSe_2_ facilitates efficient charge transfer, reducing recombination losses. The 2D morphology of MoSe_2_ provides a large surface area, enhancing dye adsorption and electron transport. Additionally, PANI contributes to superior conductivity and catalytic activity, improving redox reactions at the counter electrode. The synergistic effect between these components enhances charge separation and transport dynamics, ultimately boosting the device’s performance. I–V characterization measures the photovoltaic performance of the PANI@2D-MoSe_2_ composite, providing key parameters like efficiency, fill factor, and current density. It helps assess the composite’s effectiveness in enhancing charge transfer and overall DSSC performance.

## 4. Conclusions

In conclusion, the PANI@2D MoSe_2_ composite was successfully prepared using the electrochemical polymerization method. The surface morphology microstructure FE-SEM image analysis revealed the polymer-covered sheet-like structure of the PANI@2D MoSe_2_. The HR-TEM nanostructure surface image study also observed 2D MoSe_2_ sheet-like composites in the PANI. The X-ray diffraction pattern results confirmed electropolymerized PANI@2D MoSe_2_ in the octahedral phase. FT-IR spectroscopy analysis confirmed that the PANI containing the cyano groups was attached to molybdenum hydroxide at the regions of 1672 cm^−1^ and 3371 cm^−1^. In addition, EIS studies demonstrated that the composite CE exhibited the highest electrical conductivity of 3.47 × 10^−4^ S/cm. Finally, the electropolymerized PANI@2D MoSe_2_ composite CE achieved 7.38% efficiency. The electropolymerization of PANI@2D-MoSe_2_ ensures the precise control over film thickness, uniformity, and doping levels, enhancing conductivity and charge transfer. Unlike chemical or solvothermal methods, electropolymerization occurs under mild conditions, avoiding harsh reagents and high temperatures. This method improves interfacial adhesion, structural integrity, and electrocatalytic activity, making it ideal for DSSC applications.

## Figures and Tables

**Table 1:** I–V curve values of the as-prepared PANI, 2D-MoSe_2_, PANI@2D-MoSe_2_ composite, and Pt CEs for the DSSC.

Counter Electrode	Jsc (mA cm^−2^)	Voc (V)	FF (%)	Ƞ (%)
PANI	15.45	0.73	0.45	5.07
2D-MoSe_2_	16.09	0.77	0.47	5.82
PANI@2D-MoSe_2_	17.33	0.82	0.52	7.38
Pt	16.75	0.79	0.50	6.61

## Data Availability

The data included in this study can be made available to interested researchers upon request to the corresponding author.

## References

[1] Alessa H., Wijayantha K.G.U. (2024). A very simple flexible tandem dye-sensitized solar cell. J. Umm Al-Qura Univ. Appl. Sci..

[2] Mozaffari S., Nateghi M.R., Zarandi M.B. (2017). An overview of the Challenges in the commercialization of dye sensitized solar cells. Renew. Sustain. Energy Rev..

[3] Barichello J., Mariani P., Vesce L., Spadaro D., Citro I., Matteocci F., Bartolotta A., Di Carlo A., Calogero G. (2023). Bifacial dye-sensitized solar cells for indoor and outdoor renewable energy-based application. J. Mater. Chem. C.

[4] Tang Z., Wu J., Zheng M., Huo J., Lan Z. (2013). A microporous platinum counter electrode used in dye-sensitized solar cells. Nano Energy.

[5] Wu K., Chen L., Sun X., Wu M. (2016). Transition-Metal-Modified Polyaniline Nanofiber Counter Electrode for Dye-Sensitized Solar Cells. ChemElectroChem.

[6] Kumar R., Bhargava P. (2018). Synthesis and characterization of carbon based counter electrode for dye sensitized solar cells (DSSCs) using organic precursor 2-2′Bipyridine (Bpy) as a carbon material. J. Alloys Compd..

[7] Sarker S., Lee K.S., Seo H.W., Jin Y.K., Kim D.M. (2017). Reduced graphene oxide for Pt-free counter electrodes of dye-sensitized solar cells. Sol. Energy.

[8] Yang Z., Chen T., He R., Li H., Lin H., Li L., Zou G., Jia Q., Peng H. (2013). A novel carbon nanotube/polymer composite film for counter electrodes of dye-sensitized solar cells. Polym. Chem..

[9] Perminov A., Jurisch M., Bartzsch G., Biermann H., Weißgärber T., Volkova O. (2021). Manufacturing Fe–TiC metal matrix composite by electron beam powder bed fusion from pre-alloyed gas atomized powder. Mater. Sci. Eng. A.

[10] Ghafoor U., Aqeel A.B., Zaman U.K.U., Zahid T., Noman M., Ahmad M.S. (2021). Effect of molybdenum disulfide on the performance of polyaniline based counter electrode for dye-sensitized solar cell applications. Energies.

[11] Sheela S.E., Murugadoss V., Sittaramane R., Angaiah S. (2020). Development of tungsten diselenide/polyaniline composite nanofibers as an efficient electrocatalytic counter electrode material for dye-sensitized solar cell. Sol. Energy.

[12] Sowbakkiyavathi E.S., Murugadoss V., Sittaramane R., Angaiah S. (2020). Development of MoSe_2_/PANI composite nanofibers as an alternative to Pt counter electrode to boost the photoconversion efficiency of dye sensitized solar cell. J. Solid State Electrochem..

[13] Singh A., Poddar D., Thakur S., Jha R. (2021). Ternary composite based on MoSe_2_-rGO/polyaniline as an efficient counter electrode catalyst for dye sensitized solar cells. Mater. Chem. Phys..

[14] Yu Y., Xu A., Zhang Y., Zhao Z., Ye S., Qin Y. (2023). Construction of hierarchical graphene/polyaniline@polyaniline electrodes by chemical and electrochemical polymerization for high-energy supercapacitors. Electrochim. Acta.

[15] Mittal H., Kumar A., Khanuja M. (2022). MoSe_2_-PANI nanocomposite as Supercapacitor Electrode Material: Optimization, Mechanism and Electrochemical Performance. ChemistrySelect.

[16] Zhang H., He G., Zheng D., HuangFu H., Li Y., Mi Y., Wu M., Yuan H. (2023). Higher energy density of MoSe_2_/polyaniline capsule nanospheres for enhanced performance supercapacitor. Nanotechnology.

[17] Saha S., Chaudhary N., Mittal H., Gupta G., Khanuja M. (2019). Inorganic–organic nanohybrid of MoS_2_-PANI for advanced photocatalytic application. Int. Nano Lett..

[18] Pang Z., Chen Z., Wen R., Zhao Y., Wei A., Liu J., Tao L., Luo D., Yang Y., Xiao Y. (2017). Colloidally synthesized MoSe_2_ nano-flowers anchored on three-dimensional porous reduced graphene oxide thin films as advanced counter electrode for dye-sensitized solar cells. J. Mater. Sci. Mater. Electron..

[19] Zatirostami A. (2020). A new electrochemically prepared composite counter electrode for dye-sensitized solar cells. Thin Solid Film..

[20] Dawo C., Iyer P.K., Chaturvedi H. (2023). Carbon nanotubes/PANI composite as an efficient counter electrode material for dye sensitized solar cell. Mater. Sci. Eng. B.

[21] Mehmood U., Asghar H., Babar F., Younas M. (2020). Effect of graphene contents in polyaniline/graphene composites counter electrode material on the photovoltaic performance of dye-sensitized solar cells (DSSCSs). Sol. Energy.

[22] Velu K.S., Senthilkumaran M., Sethuraman V., Balaji M., Saravanan C., Ahmed N., Mohandoss S., Lee Y.R., Anandharaj J., Stalin T. (2023). The surfactants mediated electropolymerized poly(aniline) (PANI)-reduced graphene oxide (rGO) composite counter electrode for dye-sensitized solar cell. J. Phys. Chem. Solids.

[23] Al-bahrani M.R., Xu X., Ahmad W., Ren X., Su J., Cheng Z., Gao Y. (2014). Highly efficient dye-sensitized solar cell with GNS/MWCNT/PANI as a counter electrode. Mater. Res. Bull..

[24] Shahid M.U., Mohamed N.M., Muhsan A.S., Bashiri R., Shamsudin A.E., Zaine S.N.A. (2019). Few-layer graphene supported polyaniline (PANI) film as a transparent counter electrode for dye-sensitized solar cells. Diam. Relat. Mater..

[25] Ahmed U., Shahid M.M., Shahabuddin S., Abd Rahim N., Alizadeh M., Pandey A.K., Sagadevan S. (2021). Pandey, Suresh Sagadevan, An efficient platform based on strontium titanate nanocubes interleaved polypyrrole nanohybrid as counter electrode for dye-sensitized solar cell. J. Alloys Compd..

[26] Chen X., Liu J., Qian K., Wang J. (2018). Ternary composites of Ni–polyaniline–graphene as counter electrodes for dye-sensitized solar cells. RSC Adv..

[27] Yang P., Duan J., Tang Q. (2015). Cobalt sulfide decorated polyaniline complex counter electrodes for efficient dye-sensitized solar cells. Electrochim. Acta.

